# Influence of Hand File Size on the Accuracy of Root ZX and iPex Electronic Apex Locators: An In Vitro Study

**DOI:** 10.7759/cureus.39662

**Published:** 2023-05-29

**Authors:** Amna Y Siddiqui, Osama S Alothmani

**Affiliations:** 1 Department of Endodontics, King Abdulaziz University, Jeddah, SAU

**Keywords:** reference length, electronic apex locator measurements, ipex, root zx, hand file size, accuracy of apex locator

## Abstract

Objective: To investigate the effect of the hand file size on the accuracy of Root ZX (J. Morita Co., Kyoto, Japan) and iPex (NSK, Tochigi, Japan).

Methods: Seventy-five single-rooted teeth were decoronated, and canals were coronally flared with Gates Glidden burs sizes 4, 3, and 2. Actual canal length was determined by averaging two readings obtained by inserting K-file size 8 until its tip was apparent at the most coronal border of the apical foramen. The reference length was actual length-0.5 mm. The blinded operator utilized Root ZX and iPex following the manufacturer’s recommendations. Teeth were placed in sponge blocks soaked with Ringer’s solution. Canals were irrigated with 5% sodium hypochlorite. K-file size 8 was attached to the lip clip and introduced until the APEX/0.0 mark, then withdrawn to the 0.5 mark. A stable meter gauge for five seconds indicated an acceptable reading. Readings with sizes 10 and 15 were obtained afterward. All measurements were done twice, then averaged. Data analysis was done using ANOVA and a posthoc Bonferroni test with the significance level set at *P*<0.05.

Results: For Root ZX, the mean length with size 8 was not significantly different from the mean reference length (*P*=0.205). The same was found for its mean length at size 10 (*P*=0.093). However, the mean Root ZX length with size 15 was significantly shorter than the mean reference length (*P*=0.019). Mean iPex lengths with sizes 8, 10, and 15 were all significantly shorter than the mean reference length (*P*=0.038, 0.006, and 0.02, respectively).

Conclusion: The size of the hand file affected the precision of Root ZX and iPex.

## Introduction

Electronic apex locators (EALs) have undergone a remarkable evolution since their introduction in the early sixties. These devices, with different built-in electrical circuits and operation principles, indicate canal length after the establishment of a closed electrical circuit between the lip clip and the file attached to the EAL [1,2]. Obtaining accurate canal length will facilitate cleaning, shaping, and root filling to the optimal length, which will positively impact the success of root canal treatment. The size of the hand file used in conjunction with the EAL might impact its accuracy. Using a hand file of a size compatible with the diameter of the canal’s apical area would allow more contact between the file and the surrounding dentine, which alternatively could impact EAL function. Meanwhile, using a file size smaller than the apical diameter of the canal could allow more irrigants to fill the area between the file and dentine, which might influence the precision of the EAL [3]. 

Conflicting results have been reported regarding the suitable hand file size to be used for optimal EAL performance. The size of the hand file did not impact the precision of several EALs, including the Root ZX [4], Elements Apex Locator, Justy II, ProPex II, or Raypex 5 [5], Root ZX II, ProPex, or iPex [6], in addition to ProPex Pixi [7]. Another study reported that the use of a hand file of a size matching the enlarged canal resulted in significantly higher Root ZX accuracy when canals were filled with blood, while the effect of hand file size was diminished when canals were filled with sodium hypochlorite [8]. Others stated that the size of the hand file impacted the accuracy of Root ZX only when the diameter of the apical constriction was enlarged to 1.02 mm or when the diameter of the apical foramen exceeded 0.6 mm. Under these conditions, the use of hand files with sizes compatible with the canal’s size yielded higher accuracy compared to smaller hand files [9,10]. The accuracy of Root ZX was significantly higher when it was used with a snugly-fit hand file, while the accuracy of Novapex and Justy II was not affected by the size of the file [11]. It is worth noting that some of these studies assessed the accuracy of EALs in teeth with apical diameters purposefully enlarged to variable dimensions [4,8-10]. This approach altered the morphology of the apical constriction and the apical foramen before using the EAL. Further, the actual canal length in these studies was obtained before instrumenting the canals to large sizes. Since instrumentation shortens canal length [10,12,13], comparing EAL length to actual length in these studies might be misleading since the lengths were obtained under two different conditions: instrumented and uninstrumented canals, respectively. Regarding Root ZX, studies utilizing un-instrumented canals reported contradictory findings [6,11]. Further, our literature search revealed only one study [6] that assessed the impact of hand file size on the performance of iPex and found no significant effect, as mentioned earlier.

Given the paucity of information regarding the suitable hand file size to be used for optimum EAL function, this study aimed to investigate the effect of the hand file size on the accuracy of Root ZX (J. Morita Co., Kyoto, Japan) and iPex (NSK, Tochigi, Japan). The null hypothesis was that the accuracy of Root ZX and iPex would not be affected when used with K-file sizes 8, 10, and 15.

## Materials and methods

Seventy-five single-rooted, fully formed, extracted human teeth were included in this study. The selected teeth were free of caries and restorations. They were inspected under a dental operating microscope to confirm the presence of a closed apex and the absence of cracks or root resorption. Bucco-lingual and mesiodistal periapical radiographs were obtained to confirm the presence of a single canal. Teeth were kept in 5% sodium hypochlorite (NaOCl) for one day to remove any adherent organic tissue, and a hand scaler was used to remove calculus. Teeth were rinsed under running water, stored in numbered bottles filled with saline, and kept refrigerated until use. All teeth were decoronated at the level of the cementoenamel junction to produce a flat reference point. This was followed by coronal flaring with Gates Glidden burs sizes 4, 3, and 2 (MANI Inc., Tochigi, Japan) in a crown-down manner. Canals were irrigated with 2 mL of 5% NaOCl, with the side-vented needle inserted as deep as possible without binding.

Two blinded operators participated in data collection. Actual canal length determination was performed by the first operator for all teeth under 4X magnification by the passive introduction of K-file size 8 with double stoppers until its tip was apparent at the most coronal border of the apical foramen. The rubber stoppers were adjusted to the flat coronal reference point, and the file was withdrawn and measured using a caliper to an accuracy of 0.01 mm. This step was repeated, and then the two acquired lengths were averaged to determine the actual canal length. A deduction of 0.5 mm from the actual canal length yielded the reference length.

Teeth were placed in a plastic container filled with porous sponge blocks (Oasis Floral Foam, Kedah, Malaysia) soaked with Ringer’s solution. A pool of solution at the base of the container ensured adequate conductivity [14]. Two small holes were made in a medium dental dam sheet (one for the tooth and one for the lip clip). The lip clip and the teeth were firmly placed in the block (one tooth at a time), with the dam stretched over the assembly.

The second operator, blinded to reference lengths, measured the canals with the Root ZX and iPex following the manufacturer’s recommendations. For Root ZX, a K-file with two rubber stoppers was attached to the file clip and apically advanced until the APEX mark. The file was withdrawn until the meter gauge indicated 0.5 mm. For iPex, the file was introduced until the meter gauge indicated the 0.0 mark, then withdrawn to the 0.5 mark. The stability of the meter gauge for five seconds was mandatory to accept the readings of the EALs. If accepted, the two rubber stoppers were adjusted to the coronal reference point, and the file was withdrawn and measured as previously mentioned. Canals were irrigated with 2 mL of 5% NaOCl, as mentioned earlier. Excess irrigant was soaked with cotton pellets. The sequence of EAL utilization was randomized [11,15]. Root ZX was used before iPex in 37 randomly selected teeth, while iPex was used before Root ZX in the remaining 38 teeth. All EALs measurements were done twice, then averaged. At first, K-file size 8 was used to obtain the first reading of the first EAL for the entire sample, and it was used with the other EAL to obtain its first reading. Then the second reading was acquired for the entire sample on the first EAL, followed by the second recording on the other device. Measurements with K-file sizes 10 and 15 followed the same protocol. Measurements were done over several days. 

Microsoft Excel 365 (Redmond, USA) was used for data entry, and statistical comparisons of the mean recorded lengths were obtained by Root ZX and iPex using ANOVA and posthoc Bonferroni tests with the level of significance set at P<0.05.

## Results

For Root ZX, mean length with size 8 was not significantly different from mean reference length (P=0.205). The same was found for its mean length at size 10 (P=0.093). However, the mean Root ZX length with size 15 was significantly shorter than the mean reference length (P=0.019). Mean iPex lengths with sizes 8, 10, and 15 were all significantly shorter than the mean reference length (P=0.038, 0.006, and 0.02, respectively). Table 1 lists the mean ± standard deviation for reference length, Root ZX, and iPex with the different file sizes used. Table 2 lists the frequency of Root ZX and iPex measurements that were exact, within ± 0.5 mm and within ± 1 mm from the reference length, in relation to the different hand file sizes used.

**Table 1:** Mean ± standard deviation (mm) for reference and EALs’ lengths with different hand file sizes *Significantly shorter than the mean reference length

Measurement		Mean ± Standard Deviation (mm)
Reference length		13.19 ± 1.45
Size 8	Root ZX	12.73 ± 1.59
	iPex	12.56 ± 1.58^*^
Size 10	Root ZX	12.65 ± 1.58
	iPex	12.41 ± 1.57^*^
Size 15	Root ZX	12.5 ± 1.58^*^
	iPex	12.31 ± 1.58^*^

**Table 2:** Frequency of exact EALs’ measurements and those within ± 0.5 mm and ± 1 mm from reference length in relation to different hand file sizes used

Range of measurements in relation to reference length	Root ZX (%)			iPex (%)		
	Size 8	Size 10	Size 15	Size 8	Size 10	Size 15
Exact	7	4	7	7	4	7
± 0.5 mm	59	56	47	51	36	29
± 1 mm	84	79	68	70	59	54

## Discussion

The current in vitro study aimed to investigate whether the accuracy of Root ZX and iPex would be affected by the size of the hand file used. Results showed that the use of hand file size 15 with Root ZX significantly reduced the device’s accuracy. Root ZX recorded higher accuracy when it was used with size 8 compared to size 10. However, this difference was not statistically significant. The mean iPex length was significantly shorter than the mean reference length, regardless of the size of the hand file. The nearest iPex measurement to reference length (i.e., the least inaccurate) was obtained with size 8 (Table 1). Differences in the performance of the two devices, although they were used to measure the length of the same teeth, could be attributed to their different functioning principles. Root ZX measures the impedance ratio of two frequencies (0.4 kHz and 8 kHz) to detect the apical constriction, where the value of this ratio is 0.72 [16]. To be precise, the value of 0.72 was measured at 0.5 mm coronal to the apical foramen, a position at which the level of apical constriction was assumed but not histologically confirmed [17]. iPex utilizes multiple frequencies to simultaneously measure the resistance and capacitance to determine the position of the apical foramen [18]. Differences in length determination between the two EALs, despite being operated on the same teeth, are in line with the notion that generalization of the findings related to a certain EAL to other devices is not warranted [19].

Despite the potential differences in the electrical current passage between in vivo and in vitro conditions [20], conducting the current study in vivo would be cumbersome since multiple EAL readings were needed for each tooth (six measurements per device), followed by the need to extract the teeth to accurately determine the reference length. This would have limited the sample size. In vitro, conduction allowed testing of the EALs in a large sample size over several days, which minimized the chance for length recall and consequent operator bias. Further, in vitro EAL readings were not significantly different from those obtained in vivo [21,22]. It has been reported that the type of embedding medium had no significant effect on the accuracy of Root ZX [23]. Whether this applies to iPex is unknown since no study has evaluated the effect of different embedding media on iPex’s performance. We chose to place teeth in plastic blocks soaked with Ringer’s solution. Only one previous study utilized this model and found that 66.7% of Tri Auto ZX readings and 83.3% of Raypex 5 measurements were within ± 0.5 mm of the apical constriction [14]. This was higher than the range of readings within ± 0.5 mm reported in the current study (Table 2). This could be related to the fact that Altenburger et al. [14] used rotary files with both EALs they tested and determined the relation of the file tip to the apical constriction under a light microscope.

It is unlikely that our choice of irrigating the canals with NaOCl has influenced our results since neither the accuracy of Root ZX nor iPex was affected by the type of irrigant [24-26]. Several steps were taken to minimize the chances of bias. The sequence of EAL used was randomized [11,15]. The sequence of hand files used took into consideration the need to not introduce larger hand files before the smaller ones to control for the possible alteration in apical morphology consequent to canal enlargement. The operator who utilized the EALs was blinded to the reference length. Since each EAL measurement was repeated twice to reduce the chance of length recall, the first recording was done for the entire sample for one EAL and then for the other. This was followed by the second recording in a similar manner. In addition, measurements were done on several days.

Our results indicate that the size of the hand file has an impact on the precision of Root ZX and iPex. For Root ZX, 47%-59% of the readings were within the strict tolerance limit of ± 0.5 mm, while 68%-84% of the readings were within the more lenient tolerance limit of ± 1 mm. Corresponding iPex figures were 29%-51% and 54%-70%, respectively. Both devices recorded similar frequencies in regards to exact readings (Table 2). These findings were not in line with those of Aggarwal et al. [6]. Despite investigating the effect of larger K-file sizes (from 8 to 30), Aggarwal et al. [6] included only 10 teeth/EAL, and the distance between the file tip and the minor diameter was measured using Image J software. They reported that exact Root ZX readings ranged between 0 and 10%. A range of 57% to 70% of Root ZX readings were within the ± 0.5 mm range of accuracy, and 85% to 100% were within the ± 1 mm. The corresponding figures for iPex were 0-20%, 60-80%, and 78%-100%, respectively. The disparities between the results of the two studies could be attributed to the difference in sample size and method for determining the reference length. Further, Aggarwal et al. [6] acquired iPex lengths at the 0.0 mark of the digital display, while we chose to adopt the 0.5 mark following the manufacturer’s recommendations.

Based on Table 1, Root ZX recorded significantly more accurate readings when it was used with sizes 8 and 10 compared to size 15. Thus, we recommend using a file size that is smaller than the diameter of the canal’s apical area when operating Root ZX. This contradicted the recommendations of de Vasconcelos et al. [11], where the use of snugly-fit hand files has been advised to increase the chances of Root ZX locating the apical foramen. Whereas the sizes of the hand files to be used with the EALs were pre-defined in the current study, de Vasconcelos et al. [11] determined the sizes of the hand files to be used depending on the size of the canals to be measured. Hence, the sizes of the hand files were not standardized across the 40 teeth measured. Further, after obtaining the reference length, they opted to enlarge canals with ProFile up to size 30/06, 2 mm short of the reference length, then performed the Root ZX measurements. This might have favored the use of larger, snugly-fit hand files with the device. Further, they used the Root ZX to the APEX mark since their reference length was set at the apical foramen, unlike the approach of the current study, in which the Root ZX was used according to the manufacturer’s recommendations and the reference length was set 0.5 mm coronal to the apical foramen.

The advantage of the current study was the use of extracted teeth, which allowed for a large sample size and reduced operator bias in attaining canal measurements. Nonetheless, this research has its limitations. As mentioned earlier, different EALs have different functioning principles. Thus, generalization of the current results for other brands of EALs is not advised. Moreover, the results of this in vitro study should be interpreted with caution in in vivo situations. Further studies are needed to identify the optimum hand file size needed to facilitate the best iPex performance, while it is best to operate Root ZX with K-file sizes 8 or 10 to achieve the most accurate reading. This will facilitate a more thorough disinfection of the root canal system and the subsequent seal needed to maintain and restore periapical health.

## Conclusions

Within the limitations of the study, it seems that the differences in the electrical circuits and operation principles of different EALs are differently impacted by the size of the hand file used. Higher Root ZX accuracy was encountered when it was used with hand file sizes 8 or 10, while the use of size 15 significantly reduced its precision. For iPex, none of the three hand file sizes provided optimal readings.
